# Arctic *Micromonas* uses protein pools and non-photochemical quenching to cope with temperature restrictions on Photosystem II protein turnover

**DOI:** 10.1007/s11120-016-0310-6

**Published:** 2016-09-17

**Authors:** Guangyan Ni, Gabrielle Zimbalatti, Cole D. Murphy, Audrey B. Barnett, Christopher M. Arsenault, Gang Li, Amanda M. Cockshutt, Douglas A. Campbell

**Affiliations:** 1 Department of Chemistry & Biochemistry, Mount Allison University, 63B York St., Sackville, NB E4L3M7 Canada; 2 Key Laboratory of Vegetation Restoration and Management of Degraded Ecosystems, South China Botanical Garden, CAS, Guangzhou, 510160 China; 3 Michigan Technological University, Houghton, MI 49931 USA; 4 Key Laboratory of Tropical Marine Bio-resources and Ecology, South China Sea Institute of Oceanology, CAS, Guangzhou, 510301 China

**Keywords:** Prasinophyte, Photosystem II, Photoinactivation, Xanthophyll cycle

## Abstract

*Micromonas* strains of small prasinophyte green algae are found throughout the world’s oceans, exploiting widely different niches. We grew arctic and temperate strains of *Micromonas* and compared their susceptibilities to photoinactivation of Photosystem II, their counteracting Photosystem II repair capacities, their Photosystem II content, and their induction and relaxation of non-photochemical quenching. In the arctic strain *Micromonas* NCMA 2099, the cellular content of active Photosystem II represents only about 50 % of total Photosystem II protein, as a slow rate constant for clearance of PsbA protein limits instantaneous repair. In contrast, the temperate strain NCMA 1646 shows a faster clearance of PsbA protein which allows it to maintain active Photosystem II content equivalent to total Photosystem II protein. Under growth at 2 °C, the arctic *Micromonas* maintains a constitutive induction of xanthophyll deepoxidation, shown by second-derivative whole-cell spectra, which supports strong induction of non-photochemical quenching under low to moderate light, even if xanthophyll cycling is blocked. This non-photochemical quenching, however, relaxes during subsequent darkness with kinetics nearly comparable to the temperate *Micromonas* NCMA 1646, thereby limiting the opportunity cost of sustained downregulation of PSII function after a decrease in light.

## Introduction

*Micromonas* is a genera of small (1.5–3.0 μm) unicellular prasinophyte algae with a pear-shaped naked cell body, a single flagellum and a characteristic swimming behavior (Butcher 1952; Manton and Parke 1960). It belongs to the Mamiellales order and was the first described picoplanktonic species, initially characterized as *Chromulinapusilla* (Butcher 1952). *Micromonas* is a ubiquitous and cosmopolitan genera of picoeukaryote (Thomsen and Buck 1998), as strains occur in both near shore and oceanic environments and across a wide latitudinal temperature range (Butcher 1952; Foulon et al. 2008). In some locations, such as the coastal waters of the English Channel (Not et al. 2004) and Beaufort Sea (Lovejoy et al. 2007), *Micromonas* dominates the picoeukaryotic community throughout the year. *Micromonas* (e.g., NCMA1545 and RCC299) has a richer set of nutrient transporter gene families and contains a more complex suite of genes to counter reactive oxygen species and heavy metals as compared to the picoprasinophyte *Ostreococcus,* also a member of the Mamiellales order (Worden et al. 2009). Thus, *Micromonas* is more flexible in terms of environmental adaptability, which could explain its broader global distribution (Archibald 2009; Worden et al. 2009). Phylogenetic analysis of several genes from worldwide *Micromonas* isolations revealed three (Guillou et al. 2004) to five (Slapeta et al. 2005) phylogenetically discrete clades, suggesting this taxon is a complex of cryptic species that started to diverge during the late Cretaceous (Slapeta et al. 2005). After detecting and quantifying the genetic clades in samples from tropical, temperate and arctic environments, Foulon et al. (2008) indicated three phylogenetic clades of *Micromonas* that occupy specific niches and confirmed the existence of cryptic species within the morphospecies *Micromonas*. Lovejoy et al. (2007) then isolated and characterized the growth of a psychrophilic arctic strain of *Micromonas* NCMA 2099.

The sensitivity of Arctic plankton to warming temperatures, in parallel with higher light, is important in view of current observations and model results that the arctic is becoming warmer at much faster rates than elsewhere (Stroeve et al. 2005). As part of our wider survey of phytoplankton susceptibilities to photoinactivation of Photosystem II (Six et al. 2007, 2009; Key et al. 2010; Wu et al. 2011, 2012; Thomas and Campbell 2013; Campbell et al. 2013; Lavaud et al. 2016) we therefore sought to compare the responses of arctic and temperate strains of *Micromonas* to upward light challenges. Arctic *Micromonas* NCMA 2099 (Lovejoy et al. 2007) maintains growth at 0 °C, grows optimally at 6–8 °C, and is unable to grow above 12.5 °C. This psychrophilic strain shows light saturation of growth at or below 10 µmol photons m^−2^ s^−1^ and shows impairment of growth at higher irradiances. In contrast, temperate *Micromonas* NCMA 1646 grows optimally under warmer (18–22 °C), brighter conditions in the Mediterranean with growth-saturating light of 100 µmol photons m^−2^ s^−1^ or higher (McRose 2011). Our experiments showed that non-photochemical quenching is a major aspect of the differential responses of arctic and temperate *Micromonas* to light fluctuations.

## Materials and methods

### Culture growth and spectral measures

We cultured two strains of *Micromonas*, temperate origin NCMA 1646 at 20 °C under 36 and 185 μmol photons m^−2^ s^−1^ growth light, and arctic origin NCMA 2099 at 2 and 10 °C under 36 μmol photons m^−2^ s^−1^ growth light, in 6-well plates in a volume of 6.5 ml per well, in batch cultures in incubators. The strains were obtained from the Provasoli-Guillard National Center of Marine Phytoplankton and cultured in L1-Si media prepared using filtrated seawater according to (Keller et al. 1987; Guillard and Hargraves 1993). We used a 12:12 light/dark period and provided light from fluorescent tubes (Sylvania). The growth light was measured using a microspherical quantum sensor (US-SQS, Waltz, Germany). Cell growth was estimated using chlorophyll a fluorescence at 680 nm measured with a Molecular Devices Gemini EM spectrofluorometer. The growth rate (µ, d^−1^) was estimated as the slope of ln(Fluorescence680 nm) versus elapsed time.

Prior to each light treatment, the absorbance spectrum (a, m^−1^) from 400 to 750 nm of a culture sample was measured in a spectrophotometer (OLIS Cary 14) equipped with a DSPC integrating cavity sample chamber with an effective pathlength of ~20 cm, where near total internal reflectance within the cavity cancels light scattering resulting from suspended cells. We then extracted the cells into Mg-saturated 90 % acetone and measured chl *a* concentration (μg chl l^−1^) by absorbance (Porra 2002). The chl-specific absorption coefficient (ā*, m^−2^ mg chl^−1^) was retrieved from whole-cell absorption spectra and chl *a* (Mitchell 1990; Ciotti et al. 2002; Cai et al. 2015). We then followed (Jesus et al. 2008; Méléder et al. 2013) in the generation and interpretation of second-derivative spectra for detection of xanthophyll cycle pigments. Briefly, whole-cell spectra were normalized to the red chlorophyll *a* peak (673–675 nm) with 3–4 replicate spectra from independently grown cultures averaged for each species and treatment condition. The second derivatives of whole-cell spectra were computed with 2 nm interpolation using SpectralWorks software (OLIS). Second-derivative whole-cell spectra were normalized as in Méléder et al. (2013), using the largest negative peak from 677 to 679 nm, and 3–4 replicates of these standardized second-derivative whole-cell spectra were then averaged for each species and treatment condition in order to detect changes in xanthophyll cycle pigment content.

### Light treatments, flash yield determinations of PSII content and FRR measures

**Fig. 1:**
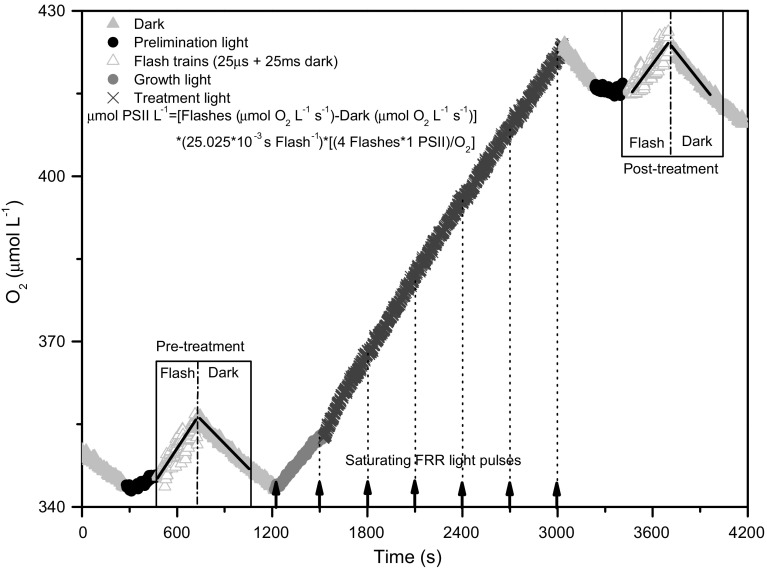
Representative O_2_ flash yield data and treatment time course. Oxygen concentration plotted versus time over a light treatment time course. The cell suspension was initially exposed to 300-s darkness for measurement of dark respiration, followed by a period of low light pre-illumination to activate photosynthesis, and then a flash train (9600 single turnover saturating flashes, each flash lasts 25 µs, interspersed by 25-ms dark) to provoke saturating single turnovers of PSII photochemistry (Chow et al. 1989; Suggett et al. 2009; Oxborough et al. 2012). In trial runs, we varied the light level and duration of the flashes to ensure they were saturating (data not shown). After the flash train, cells were again exposed to darkness and respiration immediately measured to approximate the rate of respiration prevailing during the flash train. The difference in O_2_ slope between the flash train and subsequent darkness was then used to estimate active PSII content in the sample under culture growth conditions. We then exposed cells to consecutive periods of 300 s under a treatment light level. The treatment light level was held constant through a time course of responses or, in some cases, increased in steps for a light response curve. At the end of each 300-s period, a FRR induction (Fig. 2) was applied. In parallel, we continued to use the optode to track O_2_ evolution under the treatment light levels. After the light treatment, cells were again pre-illuminated, and the O_2_ flash yield protocol was repeated to estimate active PSII center content after the light treatment. In this example, cells were arctic *Micromonas* NCMA 2099 growing at 10 °C, 36 μmol photons m^−2^ s^−1^, treated at 294 μmol photons m^−2^ s^−1^, without lincomycin

**Fig. 2:**
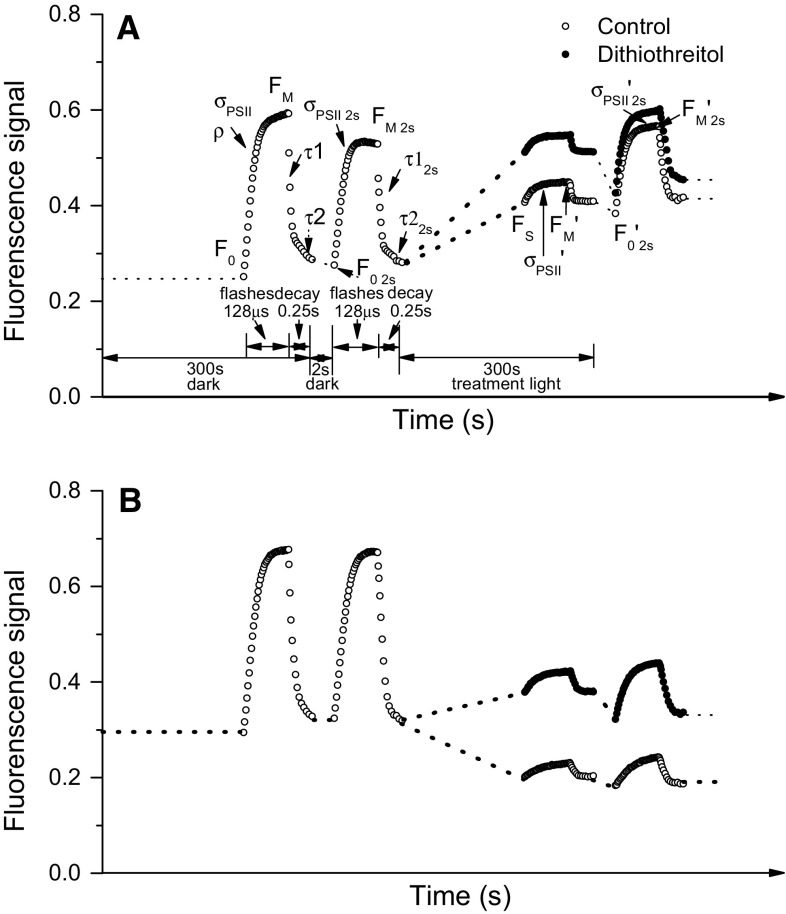
Representative chlorophyll fluorescence fast repetition rate (FRR) induction traces. **a** Temperate *Micromonas* NCMA 1646 grown at 20 °C and 185 μmol photons m^−2^ s^−1^. The initial *black dashed line* tracks a 300-s dark acclimation followed by FRR induction (*open symbol trace*) provoked by a train of 40 flashlets (1.2-μs duration, 2-μs intervening dark) applied over 128 μs that cumulatively close Photosystem II (PSII). We use a curve fit (PSIWORX-R; http://sourceforge.net/projects/psiworx/) of this initial FRR induction trace to extract the parameters *F*_0_, the basal fluorescence in the dark acclimated state, *F*_M_, the maximal fluorescence with all PSII closed, and the induction parameters *σ*_PSII_, the effective absorbance cross section serving PSII photochemistry and *ρ*, a parameter for excitation connectivity among PSII centers, in the dark acclimated state (Kolber et al. 1998; Laney 2003; Laney and Letelier 2008). Following the train of saturating flashlets, our protocol slows the flash rate, allowing PSII to reopen over a 0.25-s span. We fit this curve of PSII reopening after the saturating flash with a two-phase exponential decay to define *τ*_1_ and *τ*_2_, the fast and slow decay lifetimes reflecting electron transport processes away from PSII (Kolber et al. 1998). After a further 2-s dark period to allow reopening of PSII, we applied a second FRR induction. The *black dashed line* then spans a subsequent 300 s incubation under a treatment light (400 μmol photons m^−2^ s^−1^ in this example) ending with an FRR induction (*open symbols*) under exposure to the treatment light to define, in the light-acclimated state, *F*_S_, the fluorescence in the light-acclimated state, *F*_M_′, *σ*_PSII_′, and *ρ*′. We then again applied an FRR induction after a further 2-s dark period to allow reopening of closed PSII centers, for measurement of *F*_0_′_2s_, *F*_M_′_2s_, and *σ*_PSII_′_2s_ Note that in these cells even 2 s of darkness allows a substantial increase from *F*_M_′ to *F*_M_′_2s_, reflecting significant relaxation of non-photochemical quenching within 2 s. We used the magnitude of the increase from *F*_M_′ to *F*_M_′_2s_ to apply a proportional correction to *F*_0_′_2s_ to estimate the actual level of *F*_0_′ that prevailed under illumination, for use in subsequent parameterizations (Table 2). The *closed symbol line* tracks an FRR induction under treatment light after addition of dithiothreitol to inhibit the xanthophyll cycle deepoxidase enzyme, followed by a 2-s dark period and an additional FRR induction. **b** Arctic *Micromonas* NCMA 2099 grown at 2 °C and 36 μmol photons m^−2^ s^−1^. The measurement protocol is the same as for Fig. 2a except the 300-s light treatment was at 97 μmol photons m^−2^ s^−1^ in this example. Note that the cells show a much larger downregulation of *F*_M_ to *F*_M_′ after 300-s illumination, but that after a subsequent 2 s of darkness there is only a slight increase to *F*_M_′_2_

~30 ml of culture was harvested from 6 × 6-well plate cultures (6.5 ml per well), pooled and concentrated ~3× to 9 ml by centrifugation at 1800×*g* for 10 min followed by removal of 21 ml of media supernatant and resuspension of the cells into the remaining 9 ml. The concentrated cell suspension was then divided into three aliquots of 3 ml. A time zero (*t*_0_) sample was harvested by further centrifugation (5 min, 14,000×*g*) and then stored at −75 °C for subsequent chlorophyll analysis. A +inhibitor aliquot was created by adding either lincomycin to inhibit chloroplast protein synthesis and thereby block PSII repair (Tyystjärvi and Aro 1996) or dithiothreitol (DTT) (Bilger and Björkman 1990) to inhibit the xanthophyll deepoxidase enzyme and thereby prevent induction of xanthophyll-dependent non-photochemical quenching (NPQ). The +inhibitor and −inhibitor aliquots were loaded into 1-cm spectrophotometer cuvettes. A micro-stir bar was placed into the sample which was then sealed in with a gas-tight resin plug that incorporates a temperature control loop immersed into a 2-ml sample volume, to maintain the culture sample at its growth temperature of 2, 10 or 20 °C. The plug also incorporates a solid-state optode O_2_ sensor projecting into the sample volume with an accompanying solid-state temperature probe (FireSting system, Pyro Science GmbH, Aachen, Germany). The cuvette assembly was then placed in the Superhead optical unit of a Photon Systems Instruments FL3500 fluorometer (Brno, Czech Republic). Figures 1 and 2 and the associated legends outline the subsequent oxygen and Fast Repetition Rate fluorescence measurement and treatment protocol applied to samples. Cultures were shifted to a range of treatment light levels for (5–8) × 5 min measurement/treatment time courses. Temperate NCMA 1646 were treated at light levels from 189 to 797 μmol photons m^−2^ s^−1^, while arctic NCMA 2099 cultures were treated at light levels from 24 to 400 μmol photons m^−2^ s^−1^. Table 2 outlines the terms and definitions of photosynthetic parameters extracted directly or indirectly from the FRR induction traces using PSIWORX-R (http://sourceforge.net/projects/psiworx/) and from the time course data. Parameters for photoinactivation, repair, induction and relaxation of non-photochemical quenching were extracted from data pooled across the time course treatments at different light levels using data transform and curve fitting scripts implemented in R.

### Protein analyses

Total protein extractions were performed upon frozen cell pellets that were resuspended into 500 μl of 1× low TRIS-protein extraction buffer (50 mM TRIS buffer, 2 % lithium dodecyl sulfate, 10 % glycerol, 0.5 mM EDTA, 0.1 mg/ml 4-(2-aminoethyl) benzenesulfonyl fluoride hydrochloride (protease inhibitor)). Cells were lysed in Sigma-Aldrich bead beater tubes three times for 60 s at 4.5 m/s (MP Biomedicals Fastprep 24), with 60 s on ice between homogenization periods. The bead tubes were then centrifuged in a desktop centrifuge for 5 min at 14,800×*g*, divided into 5 aliquots of 40–50 μl and one aliquot of approximately 100 μl. These were stored at −80 °C.

Total protein concentration was determined using the BCA assay with bovine gamma globulin standards as per the manufacturer’s recommendations. Absorbance at 562 nm was measured using a Spectramax plate reader. Protein concentration of samples was quantified using the linear regression of the standard curve on triplicate samples.

For immunoquantitation (Brown et al. 2008) of PsbA, protein extracts were prepared with 0.5 μg total protein per 10 μl load. For FtsH immunoquantitations, protein extracts were prepared with 5 μg total protein in 25 or 35 μl loads. Dithiothreitol was added to a final concentration of 50 mM with the remaining sample volume made up with 1× Bolt sample buffer from Life Technologies. Standards were made by diluting stock PsbA or FtsH protein standard in the same Bolt sample buffer with a final concentration of 50 mM dithiothreitol. Samples and standards were heated at 70°C for 5 min and centrifuged to collect condensation. Prepared samples were stored at −20 °C if not used immediately.

Proteins were separated by SDS-PAGE electrophoresis in 4–12 % Bis Tris Plus 17 well polyacrylamide gels from Life Technologies. Novex Sharp Pre-stained protein standard was loaded in one lane along with MagicMark XP Immunoblot standard, both from Life Technologies. Gels were run for 30–35 min at 200 V using a Bio-Rad powerpack in 1× MES running buffer from Life Technologies. Protein was transferred from the gel to Biorad PVDF over 60 min at 20 V in 1× Bolt Transfer Buffer with Bolt Antioxidant and methanol. Following the transfer, the PVDF was blocked in 2 % ECL blocking solution made with 1× TBS-T, protein side up, on an orbital shaker, for 60 min. For PsbA, the primary antibody AS05-084 lot 1207 (AgriSera) was added in 2 % ECL blocking solution in a 1/20,000 dilution. The secondary antibody was an α-rabbit antibody, AbCam6721 in 2 % ECL blocking solution in a 1/20,000 dilution. FtsH blots used the primary antibody AS11-1789 lot 1304 (AgriSera), at a 1/5000 dilution. Secondary antibody was the same as for PsbA blots, but at a 1/5000 dilution. Each antibody incubation was 60 min, followed by a rinse cycle with 1× TBS-T: two brief rinses, one 15-min rinse, and three 5-min rinses.

**Fig. 3:**
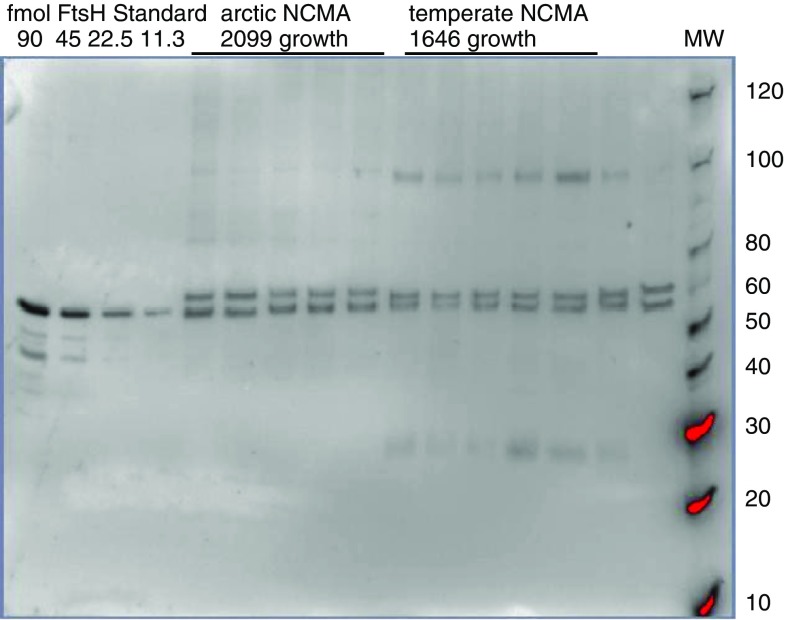
Representative blot image showing total FtsH subunit content in arctic and temperate *Micromonas*. FtsH isoforms from *Micromonas* form a double band at approximately 55 kDa.* Lanes* 1–4 contain FtsH standards (90, 45, 22.5, and 11.25 fmoles FtsH).* Lanes* 5–9 and 16 contain arctic (NCMA 2099) samples not subjected to a treatment light.* Lanes* 10–15 contain temperate (NCMA 1646) samples not subjected to a treatment light.* Lane* 17 contains MagicMark XP standard from Life Technologies. Red indicates oversaturation

Immunoblots were imaged using a Biorad Versadoc with 800 μl of ECL Select detection agent, consisting of Luminol Select and Peroxide in a 1:1 ratio. Quantitation was done using Bio-Rad Image Lab 4 for Windows. The concentration of the protein of interest was determined based on adjusted volume with a global background subtraction, and a linear or polynomial regression of the standards. A representative immunoblot image for FtsH is shown in Fig. 3.

## Results

Under low light and at 20 °C, the temperate *Micromonas* NCMA 1646 had a growth rate of 0.42 d^−1^, increasing to 1.12 d^−1^ under higher light (Table 1). The psychrophilic arctic *Micromonas* NCMA 2099 growing at 2 °C and low light grew at only 0.23 d^−1^ but achieved 0.34 d^−1^ at 10 °C, near its upper temperature limit for growth (Lovejoy et al. 2007). Thus, our arctic NCMA 2099 grew almost as fast as our temperate NCMA 1646 under comparable low light levels, but the temperate NCMA 1646 could achieve much faster growth at higher light, well above the light tolerance range of the arctic strain (data not shown). Interestingly maximum photochemical yield of PSII (*F*_V_/*F*_M_) was comparable across strains and growth conditions except for the arctic strain growing near its upper limit of 10 °C, which showed a lower *F*_V_/*F*_M_, consistent with sustained photoinhibition. The chl-specific absorption coefficient for 400 to 700 nm; m^2^ (mg chla)^−1^ (ā*) (Ciotti et al. 2002) was similar across the strains and growth conditions (Table 1).

**Table 1:** Strain information, growth and photophysiological properties

*Micromonas*	NCMA 1646		NCMA 2099	
Origin	Mediterranean		Baffin Bay	
Growth temperature (°C)	20		2	10
Growth light (μmol photons m^−2^ s^−1^)	20–36	185	20–36	20–36
Cell diameter (μm)	2–3	2–3	1–3	1–3
chl b/a	0.98 (0.04)	0.77 (0.06)	0.79 (0.01)	0.73 (0.06)
Growth rate (d^−1^)	0.42 (0.23)	1.12 (0.33)	0.23 (0.14)	0.34 (0.12)
*F*_V_/*F*_M_	0.57 (0.03)	0.56 (0.04)	0.59 (0.01)	0.49 (0.02)
*σ*_PSII_ (A^2^ quanta^−1^)	934 (66)	791 (68)	747 (52)	667 (50)
ā*, m^2^ (mg chla)^−1^	0.009 (0.001)	0.0098 (0.0005)	0.0092 (0.0004)	0.0089 (0.0004)
fmol FtsH (μg protein^−1^)	6.0 (0.7)	8.4 (0.9)	9.6 (1.8)	6.8 (0.2)
fmol PsbA (μg protein^−1^)	116 (29)	112 (28)	79 (20)	91 (5)
fmol [PSII]_active_ (μg protein^−1^)	95 (9)	133 (20)	42 (8.6)	65 (5)

*n* = 3–35, (SD)

Figures 1 and 2 and the associated legends outline the oxygen and fast repetition rate fluorescence measurement and treatment protocol applied to analyze the photophysiological responses of the *Micromonas* strains to changing light.

The temperate NCMA 1646 cells (Fig. 2a) show a significant drop from *F*_M_ measured after 300 s of dark acclimation, to *F*_M2s_ taken from the subsequent FRR induction repeated after a further 2-s dark period. This shows a rapid induction of some non-photochemical quenching (NPQ) in response to the initial FRR flashlet induction train. Over 300-s incubation at 400 μmol photons m^−2^ s^−1^, the level of *F*_0_′ increased significantly in temperate NCMA 1646 (Fig. 2a, open and closed symbols), showing significant inactivation of PSII (Ware et al. 2015a), and the level of *F*_M_′ declined significantly from the initial level of *F*_M_ showing induction of NPQ. Most of this induced NPQ_T_ relaxed after only a 2-s dark period. The presence of DTT (closed symbol trace) partially blocked the induction of NPQ, since the level of *F*_M_′ remained close to the initial *F*_M_ level, although there was still further relaxation after a 2-s dark period.

In the arctic NCMA 2099 grown at 2 °C and 36 μmol photons m^−2^ s^−1^ (Fig. 2b), the initial FRR induction protocol had limited effect upon the response to the subsequent flash after 2-s darkness, so a single induction flashlet train was not sufficient to provoke rapid induction of NPQ. The 300-s light treatment provoked a significant drop from *F*_0_ to *F*_S_, and from *F*_M_ to *F*_M2s_, showing much larger induction of NPQ than in temperate NCMA 1646 treated at 400 µmol photons m^−2^ s^−1^ (Fig. 2a). There was only slight relaxation of NPQ during a 2-s dark period. In the presence of DTT to inhibit xanthophyll deepoxidation, the overall level of fluorescence increased, reflecting a partial inhibition of NPQ. Also note the larger drop from *F*_S_ to *F*_0_′_2s_ in the presence of DTT, reflecting more PSII reaction center closure under illumination when NPQ is inhibited.

We sought to measure and compare susceptibility to photoinactivation of PSII, capacity for repair of PSII and the light and time dependencies of induction of non-photochemical quenching. In our related previous studies (Lavaud et al. 2004; Six et al. 2007, 2009; Key et al. 2010; Wu et al. 2011, 2012; Thomas and Campbell 2013; Li and Campbell 2013; Li et al. 2015; Lavaud et al. 2016), the amplitudes of induction of non-photochemical quenching were moderate and we were able to correct for any residual influence of non-photochemical quenching on the time courses of *F*_V_/*F*_M_ or *F*_V_′/*F*_M_′ which we used as proxies for changes in the function of PSII (Wu et al. 2012). In the arctic NCMA 2099, however, large and rapid induction of non-photochemical quenching, which did not fully relax within 2 s (Fig. 2b), was clearly a dominant influence on *F*_V_′/*F*_M_′, and the relaxation of non-photochemical quenching in the arctic NCMA 2099 was slow enough (20 s or greater, data not presented) as to prevent the use of *F*_V_/*F*_M_ to kinetically track changes in [PSII]_active_.

**Fig. 4:**
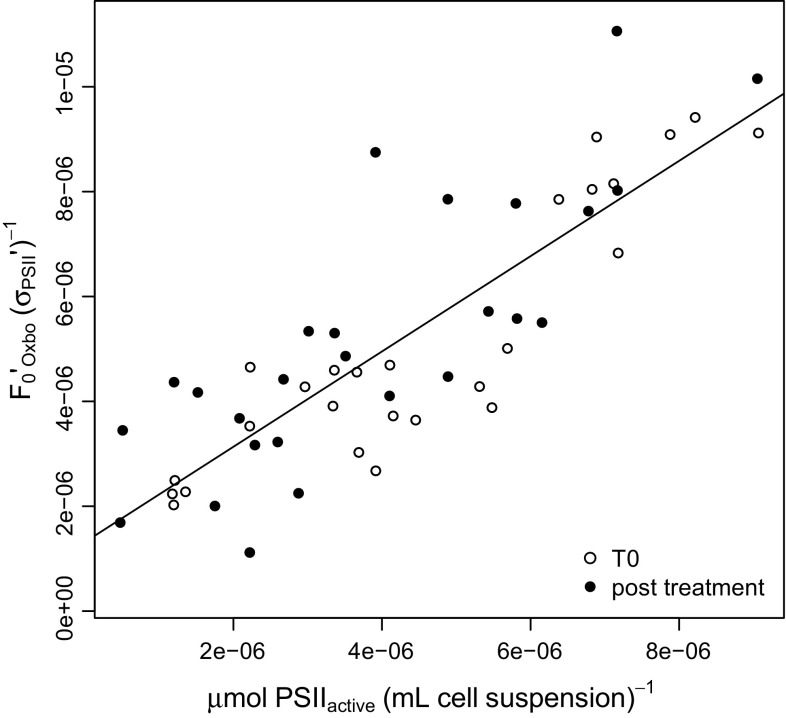
Fluorescence metric of [PSII]_active_, *F*_0_′_Oxborough_/*σ*_PSII_′ plotted versus [PSII]_active_ (µmol ml^−1^) determined from oxygen flash yields (Fig. 1). *Open symbols* show samples measured directly from growth conditions. *Closed symbols* show samples measured after a high-light treatment to induce some photoinhibition, with or without the presence of lincomycin to block PSII repair. Pooled linear regression with a slope 0.9088, intercept 1.317 × 10^−6^ and *R* ^2^ of 0.7139

We therefore sought an alternate, rapid, noninvasive measure of [PSII]_active_ to track photoinactivation and repair. Oxborough et al. (2012) and Silsbe et al. (2015) introduced *F*_0_′/*σ*_PSII_′ as a rapid measure of [PSII]_active_ with calibration against slower oxygen flash yield measures (Chow et al. 1989; Suggett et al. 2009) of [PSII]_active_. We found good correlation between *F*_0_′/*σ*_PSII_′ and oxygen flash yield measures of [PSII]_active_ for culture samples taken direct from growth conditions. This useful correlation, however, diverged after photoinhibition time courses, because photoinhibition causes a rise in *F*_0_′ (Ware et al. 2015a, b) through an increase in the fluorescence yield of photoinactivated PSII, unrelated to any increase in [PSII]_active_. Oxborough and Baker (1997) derived an estimator of *F*_0_′ (Table 2) that corrects for the influence of non-photochemical quenching on *F*_0_, but which excludes the cumulative influence of photoinactivation. We therefore plotted *F*_0_′_Oxborough_/*σ*_PSII_′ versus oxygen flash yield measures of [PSII]_active_ measured on the same samples, and found a correlation that was robust in the face of accumulated photoinactivation of PSII (Fig. 4); compare the open symbols showing measurements of samples taken directly from growth conditions, with the closed symbols showing measurements of samples after a high-light treatment to induce some photoinhibition, with or without the presence of lincomycin to block PSII repair. The data were fit with a pooled regression of slope 0.9088, intercept 1.317 × 10^−6^ and *R* ^2^ of 0.7139. In other work (Murphy et al. 2016) we are now extending this proxy for [PSII]_active_ to other species and growth conditions.

**Table 2:** Parameters and equations

Parameter	Equation	Definition, units	Reference
*F*_0_		Minimal fluorescence with PSII open	van Kooten and Snel (1990)
*F*_M_		Maximal fluorescence with PSII closed	van Kooten and Snel (1990)
*F*_S_		Fluorescence at an excitation level	van Kooten and Snel (1990)
*F*_M_′		Maximal fluorescence with PSII closed in at an excitation level	van Kooten and Snel (1990)
*F*_M_′_2s_		Maximal fluorescence with PSII closed 2 s after excitation	Fig. 2
*F*_0_′	*F*_0_′_2s_ × {1 − [(*F*_M_′_2s_ − *F*_M_ ′)/*F*_M_′_2s_]}	Minimal fluorescence with PSII open, estimated for cells under excitation, including influence of photoinactivation	van Kooten and Snel (1990), Fig. 2
*F*_0_′_2s_		Minimal fluorescence with PSII open 2 s after excitation	Fig. 2
*F*_0_′_Oxborough_	1/{(1/*F*_0_ − 1/*F*_M_ + 1/*F*_M_′)	Minimal fluorescence with PSII open, estimated for cells under excitation, excluding influence of photoinactivation	Oxborough and Baker (1997) and Ware et al. (2015a, b)
*ρ*		Excitation connectivity among PSII centers	Kolber et al. (1998)
*σ*_PSII_		Functional absorbance cross section for PSII photochemistry	Kolber et al. (1998)
*σ*_PSII_′		Functional absorbance cross section for PSII photochemistry under excitation	Kolber et al. (1998)
*σ*_PSII_′_2s_		Functional absorbance cross section for PSII photochemistry 2 s after excitation	Fig. 2
*τ*_1_		Slow lifetime for PSII reopening after saturating flash, s	Kolber et al. (1998)
*τ*_2_		Fast lifetime for PSII reopening after saturating flash, s	Kolber et al. (1998)
NPQ	(*F*_M_ − *F*_M_′)/*F*_M_′	Non-photochemical quenching	Genty et al. (1989), Kramer et al. (2004) and Klughammer and Schreiber (2008)
Y(PSII) (=*Φ*_PSII_)	(*F*_M_′ − *F*_S_)/*F*_M_′	Quantum yield for PSII photochemistry	Genty et al. (1989), Kramer et al. (2004) and Klughammer and Schreiber (2008)
Y(NO)	Y(NO) = *F*_s_/*F*_M_	Quantum yield for non-regulated non-photochemical excitation dissipation	Genty et al. (1989), Kramer et al. (2004) and Klughammer and Schreiber (2008)
Y(NPQ)	*F*_S_/*F*_M_′ − *F*_s_/*F*_M_	Quantum yield for regulated non-photochemical excitation dissipation	Genty et al. (1989), Kramer et al. (2004) and Klughammer and Schreiber (2008)
q_P_	(*F*_M_′ − *F*_S_)/(*F*_M_′ − *F*_0_′)	Photochemical quenching of fluorescence ~ fraction of open PSII	van Kooten and Snel (1990)
k_pi_	$\left[ {\text{PSII}} \right]_{\text{active}} t = \left[ {\text{PSII}} \right]_{\text{active}} t_{0 } * \, e^{{( - {\text{k}}_{\text{pi}} *t)}}$	First-order rate constant for photoinactivation of PSII, s^−1^	Kok (1956)
*σ*_i_	$$\left[ {\text{PSII}} \right]_{\text{active}} t = \left[ {\text{PSII}} \right]_{\text{active}} t_{0 } * {\text{e}}^{{( - \sigma_{\text{i}} *t*I)}}$$	Target size for photoinactivation of PSII across multiple excitation levels I, m^2^ photon^−1^	Oliver et al. (2003), Key et al. (2010) and Campbell and Tyystjärvi (2012)
k_rec_	$\left[ {\text{PSII}} \right]_{\text{active}} t = \left[ {\text{PSII}} \right]_{\text{active}} t_{0 } * ({\text{k}}_{\text{rec}} + ({\text{k}}_{{{\text{pi}} }} * {\text{e}}^{{ - ({\text{k}}_{\text{pi}} + {\text{k}}_{\text{rec}} )*t)}} ))/({\text{k}}_{\text{pi}} + {\text{k}}_{\text{rec}} )$	First-order rate constant for recovery of photoinactivated PSII, s^−1^	Kok (1956)
k_recinact_	$\left[ {\text{PSII}} \right]_{\text{active}} t = \{ \left[ {\text{PSII}} \right]_{\text{active}} t_{0 } * (({\text{k}}_{\text{recinact}} /({\text{k}}_{\text{pi}} + {\text{k}}_{\text{recinact}} )) + (({\text{k}}_{\text{pi}} /({\text{k}}_{\text{pi}} + {\text{k}}_{\text{recinact}} ))*{\text{e}}^{{ - \left( {{\text{k}}_{\text{pi}} + {\text{k}}_{\text{rec}} } \right)*t)}} )\} + \{ \left[ {\text{PSII}} \right]_{\text{inactive}} t_{0} * \, ({\text{k}}_{\text{recinact}} /({\text{k}}_{\text{pi}} + {\text{k}}_{\text{recinact}} )) \, *(1 - {\text{e}}^{{ - \left( {{\text{k}}_{\text{pi}} + {\text{k}}_{\text{rec}} } \right)*t)}} )\}$	First-order rate constant for recovery of photoinactivated PSII, allowing for initial pool of [PSII]_inactive_ *t*_0_, s^−1^	
ke_qp_	1 − I/(ke_qp_ + I)	Half-saturation light level for photochemical quenching, μmol photons m^−2^ s^−1^	
ke_npq_	$1 - {\text{Y}}\left( {\text{NPQ}} \right) = \{ (I/{\text{ke}}_{\text{npq}} + I) * {\text{e}}^{{( - {\text{k}}_{\text{npq}} *{\text{time}})}} \} + \{ 1 - [(I/{\text{ke}}_{\text{npq}} + I) - {\text{k}}_{\text{npqslow}} *{\text{cumulative time}}]\}$	Half-saturation light level for induction of non-photochemical quenching, μmol photons m^−2^ s^−1^	
k_npq_	$1 - {\text{Y}}\left( {\text{NPQ}} \right) = \{ (I/{\text{ke}}_{\text{npq}} + I) * {\text{e}}^{{( - {\text{k}}_{\text{npq}} *{\text{time}})}} \} + \{ 1 - [(I/{\text{ke}}_{\text{npq}} + I) - {\text{k}}_{\text{npqslow}} *{\text{cumulative time}}]\}$	First-order rate constant for induction of non-photochemical quenching, s^−1^	
k_npqslow_	$1 - {\text{Y}}\left( {\text{NPQ}} \right) = \{ (I/{\text{ke}}_{\text{npq}} + I) * {\text{e}}^{{( - {\text{k}}_{\text{npq}} *{\text{time}})}} \} + \{ 1 - [(I/{\text{ke}}_{\text{npq}} + I) - {\text{k}}_{\text{npqslow}} *{\text{cumulative time}}]\}$	Zero-order rate constant for time dependent induction of slow phase of non-photochemical quenching	
kr_npq_		First-order rate constant for relaxation of non-photochemical quenching, s^−1^	
ā*		The chl-specific absorption coefficient for 400 to 700 nm; m^2^ (mg chla)^−1^	Ciotti et al. (2002)
PSII_ETR_	*σ*_PSII_/(*F*_V_/*F*_M_) * Y(PSII) * *I*	PSII electron transport rate e-PSII^−1^s^−1^	Suggett et al. (2003, 2009) and Huot and Babin (2010)

**Fig. 5:**
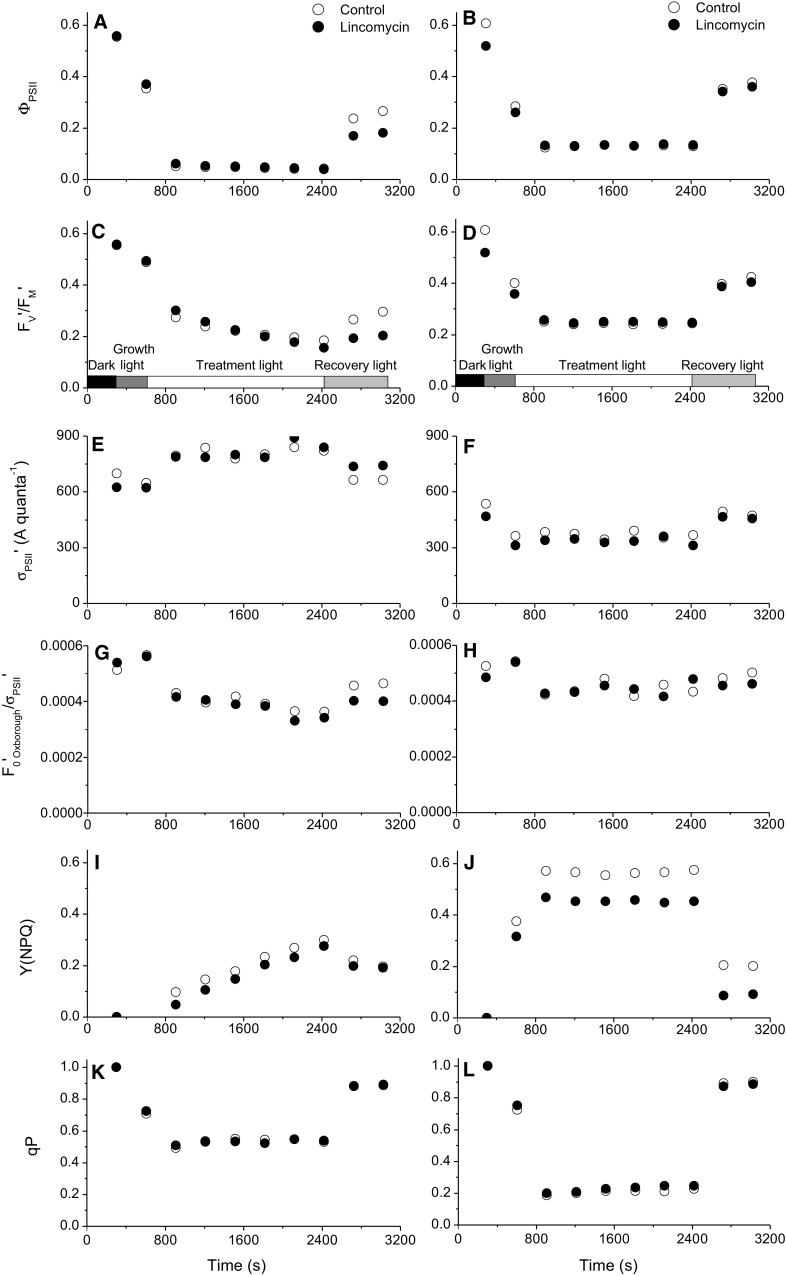
Representative time course data of PSII function from *Micromonas* cultures shifted from growth to higher light, with or without PSII repair. Temperate *Micromonas* NCMA 1646 was grown at 20 °C and 185 μmol photons m^−2^ s^−1^, and treated at 400 μmol photons m^−2^ s^−1^. Arctic *Micromonas* NCMA 2099 was grown at 2 °C and 36 μmol photons m^−2^ s^−1^ and treated at 97 μmol photons m^−2^ s^−1^. Cells were initially exposed to 300 s of dark, followed by 300 s at their growth light, and then 6 (arctic) or 7 (temperate) consecutive periods of 300 s under the treatment light. We then exposed the cells to low recovery light of 12 μmol photons m^−2^ s^−1^ for 300 s. At the end of each 300-s period, we applied the FRR induction protocol outlined in Fig. 2, of an FRR induction applied under illumination followed by 2 s of darkness and a second FRR induction. Cells then progressed to their next 300 s of light incubation. Plotted photosynthetic parameters were extracted from these FRR inductions. Data from cells treated with lincomycin to block PSII repair plotted in *closed black symbols*; data from control cells without lincomycin plotted in *open symbols*. **a**, **b** A light-induced decline in Y(PSII) (or *Φ*_PSII_) (Genty et al. 1989; Kramer et al. 2004; Klughammer and Schreiber 2008) was saturated within the first 300 s of the treatment light, with little detectable effect of lincomycin except during the low light recovery, where control temperate NCMA 1646 (**a**, *open symbols*) recovered more than lincomycin cells (**a**, *closed symbols*). **c**, **d** *F*_V_′/*F*_M_′, the maximal photochemical yield in the light-acclimated state. The difference between control and lincomycin treatments shows the influence of the PSII repair cycle on PSII function over time in the temperate cells (**a**). PSII repair had only a slight influence on the time course in arctic NCMA 2099 (**d**), with a marginally larger recovery of *F*_V_′/*F*_M_′ in the control cells (**d**, *open symbols*) compared to lincomycin-treated cells (**d**, *closed symbols*). **e**, **f** *σ*_PSII_′ is the effective absorbance cross section for PSII photochemistry measured in the light-acclimated state. Control temperate cells show a steady *σ*_PSII_′ (**e**, *open symbols*), whereas lincomycin-treated cells show some increase in *σ*_PSII_′ (**e**, *closed symbols*). Arctic NCMA 2099 shows a strong downregulation of *σ*_PSII_′ in both control and lincomycin treatments (**f**). **g**, **h** *F*_0_′_Oxborough_/*σ*_PSII_′ as an index of [PSII]_active_. **i**, **j** Y(NPQ) is the quantum yield of non-photochemical energy dissipation (Kramer et al. 2004; Klughammer and Schreiber 2008). **k**, **l** Fraction of open PSII (q_P_)

In the current study, we measured time courses (representative data in Fig. 5a–l) of PSII function measured under different treatment light levels, in the presence or absence of lincomycin to block PSII repair or DTT to block xanthophyll cycling. We used the parameter *F*_0_′_Oxborough_/*σ*_PSII_′ (Fig. 5g, h) as a proxy for the content of [PSII]_active_ (Fig. 4) to fit estimates of *σ*_i_, a target size parameterization of the susceptibility of *Micromonas* to photoinactivation of PSII (Tables 2, 3). *σ*_i_ is based upon the assumption that, at least up to moderately high light, photoinactivation is a linear product of cumulative photon dose (Oliver et al. 2003; Campbell and Tyystjärvi 2012). For this data set, for each combination of growth condition and strain we fit the set of time course measurements of *F*_0_′_Oxborough_/*σ*_PSII_′ measured under different treatment lights with a pooled *σ*_i_ (Table 3). *σ*_i_ was comparable within confidence intervals at 1 to 1.4 × 10^−24^ m^2^ photon^−1^ across the strains and growth conditions, with the intriguing exception of arctic NCMA 2099 grown at 2 °C, which showed a significantly higher *σ*_i_ of 3.6 × 10^−24^ m^2^ photon^−1^ (Table 3) indicating a higher susceptibility to photoinactivation under that growth condition. We speculate that at low growth temperature *Micromonas* suffers increased ROS toxicity under excess light, leading to increased susceptibility to photoinactivation at a given photon dose (Vass 2011, 2012).

**Table 3:** Photosystem II functional parameters from curve fitting (95 % CI)

Parameter	Temperate NCMA 1646		Arctic NCMA 2009	
Growth temperature (°C)	20	20	2	10
Growth light (μmol photons m^−2^ s^−1^)	20–36	185	20–36	20–36
n	12	16	35	14
Dark *τ*_1_ (s)	1.3 × 10^−2^ (1.3 × 10^−3^)	1.0 × 10^−2^ (0.0014)	8.4 × 10^−3^ (1 × 10^−3^)	1.3 × 10^−2^ (1.4 × 10^−3^)
Dark *τ*_2_ (s)	3 × 10^−4^ (1 × 10^−5^)	3 × 10^−4^ (1 × 10^−5^)	5.8 × 10^−4^ (7.3 × 10^−5^)	5 × 10^−4^ (1.7 × 10^−5^)
Growth light *τ*_1_ (s)	8.6 × 10^−3^ (4.6 × 10^−4^)	6 × 10^−3^ (1 × 10^−3^)	1.1 × 10^−2^ (2.9 × 10^−3^)	9.5 × 10^−3^ (2.4 × 10^−3^)
Growth light *τ*_2_ (s)	5.5 × 10^−4^ (2.3 × 10^−5^)	4.3 × 10^−4^ (2.8 × 10^−5^)	1.2 × 10^−3^ (7.3 × 10^−5^)	9 × 10^−4^ (8.2 × 10^−5^)
*σ*_i_ (m^2^ photon^−1)^	1.4 × 10^−24^ (4 × 10^−25^)	1.3 × 10^−24^ (1.4 × 10^−25^)	3.6 × 10^−24^ (5 × 10^−25^)	1 × 10^−24^ (4.3 × 10^−25^)
k_rec_ (s^−1^)	2 × 10^−4^ (2 × 10^−4^)	5 × 10^−4^ (9 × 10^−5^)	2.7 × 10^−4^ (9 × 10^−5^)	9.2 × 10^−5^ (2.5 × 10^−4^)
k_recinact_ (s^−1^)	2 × 10^−4^ (9 × 10^−5^)	5 × 10^−4^ (9 × 10^−5^)	7 × 10^−5^ (1.5 × 10^−5^)	2 × 10^−5^ (4.8 × 10^−5^)
ke_qp_ (μmol photons m^−2^ s^−1^)	145 (8)	228 (11)	64 (4)	159 (13)
DTT ke_qp_ (μmol photons m^−2^ s^−1^)	56 (11)	103 (12)	59 (6)	97 (5)
ke_npq_ (μmol photons m^−2^ s^−1^)	*2616 (6500)*	166 (70)	49 (8)	159 (53)
k_npq_ (s^−1^)	0.02 (3.1)	0.0015 (0.0003)	0.003 (0.001)	0.003 (0.0007)
k_npqslow_	<1 × 10^−5^	4.1 × 10^−5^ (1.5 × 10^−5^)	2 × 10^−6^ (9 × 10^−6^)	2 × 10^−6^ (8 × 10^−6^)
kr_npq_ (s^−1^)	1 × 10^−7^ (1 × 10^−4^)	4.1 × 10^−4^ (6 × 10^−5^)	3 × 10^−4^ (5 × 10^−5^)	5.6 × 10^−4^ (2 × 10^−4^)
DTT ke_npq_ (μmol photons m^−2^ s^−1^)	n.d.	n.d.	39 (14)	1495 (575)
DTT k_npq_ (s^−1^)	n.d.	n.d.	0.002 (0.0003)	>*0.07 (214,835)*
DTT k_npqslow_	n.d.	n.d.	<2 × 10^−5^	1.1 × 10^−5^ (2.2 × 10^−5^)

Italic values indicate poorly constrained value

Using *σ*_i_ estimated in the presence of lincomycin as an input, we then estimated k_rec_, s^−1^, a first-order rate constant for functional recovery of photoinactivated PSII (Kok 1956) using the time/light courses of *F*_0_′_Oxborough_/*σ*_PSII_′ measured in the absence of lincomycin (Fig. 5g, h), with PSII repair active (Table 3). Temperate NCMA 1646 showed an upregulation of k_rec_ with an increase in growth light at 20 °C (Table 3). To our surprise under comparable growth lights of 20–36 μmol photons m^−2^ s^−1^, the fitted k_rec_ in arctic NCMA 2099 at 2 °C was comparable to the fitted k_rec_ in temperate NCMA 1646 at 20 °C, implying that at least under low growth light and moderate treatment lights the arctic NCMA 2099 was able to maintain an active membrane-based PSII repair cycle (Nixon et al. 2010; Komenda et al. 2012), which is blocked by addition of lincomycin to inhibit PsbA translation.

The k_rec_ formulation assumes that at *t*_0_ of the time course all PSII is in the form [PSII]_active_, and that the subsequent accumulation of [PSII]_inactive_ during the light treatment generates the key substrate for PSII repair. We know that the PSII repair cycle involves multiple intermediates (Tyystjärvi et al. 2005; Nixon et al. 2010; Komenda et al. 2012). If growing cells contain an initial pool of [PSII]_inactive_ *t*_0_, this leads to an overestimation of k_rec_ under the simple Kok model (Kok 1956).

**Fig. 6:**
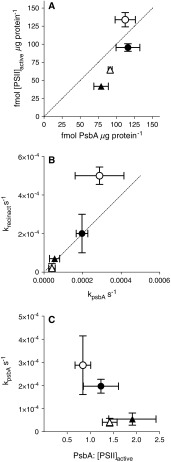
Photosystem II content and function. **a** Content of [PSII]_active_ versus PSII protein subunit PsbA. *Dotted line* indicates 1:1 ratio. **b** Functional rate constant for repair of [PSII]_inactive_ (k_recinact_; Table 3) versus rate constant for removal of PsbA protein. *Dotted line* indicates 1:1 ratio. **c** Rate constant for removal of PsbA protein versus the ratio of PsbA: [PSII]_active_. *Filled circle*, *Micromonas* NCMA 1646 grown at 20 °C and 36 μmol photons m^−2^ s^−1^. *Open circle*, *Micromonas* NCMA 1646 grown at 20 °C and 185 μmol photons m^−2^ s^−1^. *Filled triangle*, *Micromonas* NCMA 2099 grown at 2 °C and 36 μmol photons m^−2^ s^−1^. *Open triangle*, *Micromonas* NCMA 2099 grown at 10 °C and 36 μmol photons m^−2^ s^−1^. Mean of *n* = 3–4 determinations from separate cultures; ±SEM

In Fig. 6a, we compare the content of [PSII] _active_ measured using oxygen flash yields with the content of the PsbA protein subunit from Photosystem II determined by quantitative immunoblotting from the same culture samples (Fig. 6a). The arctic strain NCMA 2099 growing at 2 °C (closed triangle) contained only 42 fmol [PSII]_active_ (μg protein^−1^), compared to 79 fmol PsbA (μg protein^−1^). Therefore, ~37 fmol PsbA (μg protein^−1^) were in the form of [PSII]_inactive_, before the start of any light treatment (Fig. 5). In marked contrast, the temperate strain NCMA 1646 growing at 20 °C and 185 µmol photons m^−2^ s^−1^ (open circle) contained 133 fmol [PSII]_active_ (μg protein^−1^), equivalent within error bars to the cellular content of 112 fmol PsbA (μg protein^−1^). Therefore, in comparison with the arctic strain, the temperate strain allocated two to three times as much of its protein to [PSII]_active_, with only a negligible pool of [PSII]_inactive_ under growth conditions.

We therefore fit our data with an alternate formulation of k_recinact_ (Tables 2, 3; Fig. 6b) that estimates the rate constant for repair of Photosystem II after allowing for an initial pool of [PSII]_inactive_ *t*_0_. We used the difference between growth [PSII]_active_ and growth PsbA content as our estimator for the initial content of [PSII]_inactive_. For the arctic NCMA 2099, these fitted k_recinact_ (Table 3) were indeed three- to fourfold smaller than the simpler (Kok 1956) k_rec_ fits (Table 3), while for the temperate strains k_rec_ and k_recinact_ were similar because those cultures contained little [PSII]_inactive_ *t*_0_ under growth conditions. Furthermore the estimates of k_recinact_ accord well with experimentally independent estimates of the rate constant for clearance of PsbA protein, k_psbA_ (Fig. 6b). In parallel with our analyses of PSII function and content, we measured the abundance of the total pool of FtsH protease subunits (Table 1), some of which mediate progressive proteolytic degradation of the PsbA protein, catalyzing this rate limiting step (Nixon et al. 2010; Komenda et al. 2012; Campbell et al. 2013). The arctic cells at 2 °C had both the highest protein allocation to FtsH, and the highest ratio of FtsH/PsbA, suggesting regulation of a key enzyme content to partially counter the strong kinetic restriction on PsbA clearance at low temperature (Fig. 6b; triangles).

To summarize the time and light response of PSII closure (q_P_) (Fig. 5k, l), we used a Michaelis–Menten formulation to define a half-saturation light level for q_P_, ke_qp_, fit across time courses measured at different treatment light levels for each combination of strain and growth condition. As expected, the temperate NCMA 1646 at 20 °C responded to increased growth light with an increase in ke_qp_ from 145 to 228 μmol photons m^−2^ s^−1^ as the cells acclimated with decreased light capture through a smaller *σ*_PSII_ (A^2^ quanta^−1^) (Table 2). The arctic NCMA 2099 at 2 °C showed a low ke_qp_ of 64 μmol photons m^−2^ s^−1^, reflecting low temperature restrictions on metabolic consumption of reductant, as shown by long life times *τ*_1_ and *τ*_2_ (s) for removal of electrons from PSII under growth light (Table 3). Arctic NCMA 2099 at 10 °C largely escaped from this temperature restriction on metabolism as *τ*_1_ and *τ*_2_ under growth light decreased to ranges comparable to the temperate NCMA 1646 at 20 °C and ke_qp_ increased to 159 μmol photons m^−2^ s^−1^, comparable to the temperate NCMA 1646 at 20 °C and the same growth light level.

To test the importance of xanthophyll pigment cycling to mediate excitation dissipation, we used the inhibitor dithiothreitol (DTT) (Bilger and Björkman 1990). Addition of DTT sharply decreased ke_qp_ for the temperate NCMA 1646 and for the arctic NCMA 2099 at 10 °C, showing that loss of ongoing xanthophyll cycling lowered the flux of excitation into non-photochemical paths and increased closure of PSII. Arctic NCMA 2099 at 2 °C in contrast showed no change in ke_qp_ in response to DTT so that blockage of ongoing xanthophyll cycling did not measurably affect immediate excitation pressure upon PSII. These distinctions are illustrated qualitatively in Fig. 2, where addition of DTT to temperate NCMA 1646 results in a large closure of PSII under illumination that relaxes upon 2 s of darkness (compare dark +lincomycin trace to open −lincomycin trace, Fig. 2a). In contrast, addition of DTT to arctic NCMA 2099 provokes only moderate additional PSII closure (compare dark +lincomycin trace to open −lincomycin trace, Fig. 2b). In both the arctic and temperate strains, addition of DTT causes an increase in fluorescence levels, consistent with blockage of some non-photochemical quenching.

Induction of non-photochemical quenching in our treatments followed more complex kinetics than the simple, near-instantaneous light dependence of q_P_. We therefore fit the time/light courses of Y(NPQ) (Fig. 5i, j) (Kramer et al. 2004; Klughammer and Schreiber 2008) with a more complex equation (Table 2). We chose the Y(NPQ) formulation because it is bounded between 0 and 1, rather than the unbounded Stern–Volmer NPQ formulation (*F*_M_ − *F*_M_′)/*F*_M_′). We parameterized the amplitude of Y(NPQ) at a given light level again using a Michaelis–Menten formulation with a half-saturation light level ke_npq_. We captured the rate of approach to this Y(NPQ) amplitude using a first-order rate constant (s^−1^) for induction of non-photochemical quenching. We also observed a slower phase that accumulated as a linear function of cumulative time under irradiance (Fig. 5i, j), particularly in the temperate NCMA 1646 and in arctic NCMA 2099 when grown at 10 °C. We therefore included k_npqslow_ as a zero-order rate constant of cumulative time, accumulating a slow induction phase of Y(NPQ) (Table 2). Given the effect of DTT upon the temperate strain and upon the arctic strain growing at 10 °C, and the limited DTT effect upon the arctic strain growing at 2 °C, we suspect this k_npqslow_ zero-order induction rate represents induction of xanthophyll cycling.

The temperate NCMA 1646 at 20 °C and 20–36 μmol photons m^−2^ s^−1^ growth light showed a limited amplitude for Y(NPQ) which did not saturate under our range of treatment light levels, shown by a poorly constrained ke_npq_ of 2616 μmol photons m^−2^ s^−1^. In contrast, at 185 μmol photons m^−2^ s^−1^ growth light, temperate NCMA 1646 demonstrated stronger induction of Y(NPQ) with a ke_npq_ of 166 μmol photons m^−2^ s^−1^, already showing half saturation of Y(NPQ) induction at the culture growth light. The arctic NCMA 2099 grown at 2 °C and 20–36 μmol photons m^−2^ s^−1^ had a ke_npq_ of 49 μmol photons m^−2^ s^−1^, again showing half-saturation of Y(NPQ) induction near growth light levels, consistent with findings from ice algae (Petrou et al. 2010). Under similar growth light conditions but at a higher temperature (10 °C), the arctic NCMA 2099 showed a more gradual induction of NPQ with a higher ke_npq_ of 159 μmol photons m^−2^ s^−1^. Addition of DTT had little effect upon ke_npq_ in the arctic NCMA 2099 at 2 °C but greatly suppressed light induction of Y(NPQ) at 10 °C driving ke_npq_ up to 1495 μmol photons m^−2^ s^−1^. Y(NPQ) induction was therefore not directly dependent upon sustained xanthophyll cycling in arctic NCMA 2099 at 2 °C, but was during growth at the higher temperature. Addition of lincomycin caused a significant drop in Y(NPQ) in arctic NCMA 2099, consistent with findings (Bachmann et al. 2004; Lavaud et al. 2016) that accumulation of NPQ depends directly or indirectly upon sustained chloroplastic protein synthesis.

**Fig. 7:**
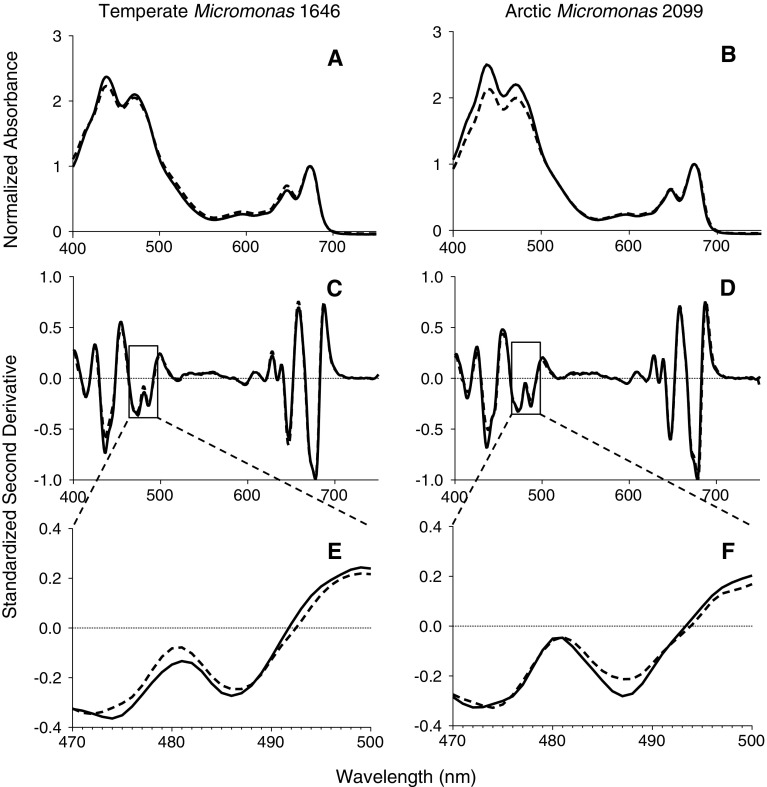
Whole-cell spectra. **a**, **b** Whole-cell visible spectra from temperate *Micromonas* NCMA 1646 (**a**) or arctic *Micromonas* NCMA 2099 (**b**) taken directly from growth conditions and captured in an integrating cavity to cancel cell suspension scattering of light. Spectra were normalized to the red chlorophyll peak before averaging. Consistent with the determinations of ā*, the whole-cell spectra were similar across strains and growth conditions. Averaged spectra of similar maximum OD from 3 separate cultures presented, 95 % CI omitted for clarity. Solid lines are the 185 μmol photons m^−2^ s^−1^ growth condition for temperate NCMA 1646 (**a**, **c**, **e**) and the 2 °C growth condition for arctic NCMA 2099 (**b**, **d**, **f**). *Dashed lines* are 36 μmol photons m^−2^ s^−1^ growth condition for NCMA 1646 (**a**, **c**, **e**) and the 10 °C growth condition for arctic NCMA 2099 (**b**, **d**, **f**). **c**, **d** Second-derivative spectra to detect inflection points of spectra from temperate NCMA 1646 (**c**) or arctic NCMA 2099 (**d**). *Solid* and *dashed lines* nearly overlay, except from 470 to 500 nm. **e**, **f** Second-derivative spectra from 470 to 500 nm in the carotenoid region from temperate NCMA 1646 (**e**) or arctic NCMA 2099 (**f**). Note the statistically significant difference (*p* < 0.05) at 487–488 nm between arctic NCMA 2099 grown at 2 °C (*solid line*) versus 10 °C (*dashed line*), reflecting differences in xanthophyll cycle pigment contents

We suspected the lack of a DTT effect upon arctic NCMA 2099 growing at 2 °C resulted from full pre-induction of xanthophyll deepoxidation in these cells before the onset of any light treatment. Therefore, in Fig. 7a we present normalized, averaged whole-cell spectra captured from the four combinations of strain and growth. From these whole-cell spectra, we extracted (Fig, 7b, c) second-derivative spectra to detect inflection points of spectra. In the second-derivative spectra from 470 to 500 nm in the carotenoid region, we detected a statistically significant difference at 487 nm between arctic NCMA 2099 grown at 2 °C versus 10 °C, likely reflecting differences in xanthophyll cycle pigment contents of zeaxanthin (485 nm) and lutein (494 nm) (Jesus et al. 2008; Six et al. 2009; Méléder et al. 2013) between these growth conditions.

After high-light exposure, we measured the relaxation of Y(NPQ) as kr_npq_, (s^−1^), a first-order rate constant (Table 3). The arctic NCMA 2099 at 2 or 10 °C showed a kr_npq_ similar within confidence intervals to the kr_npq_ fitted for the temperate NCMA 1646 at 20 °C. Thus, even though the DTT susceptibility, and thus dependence upon ongoing xanthophyll cycling, was distinct for the arctic NCMA 2099 at 2 °C, it retained the flexibility to relax NPQ on timescales comparable to the temperate NCMA 1646. Finer scale comparisons of relaxation over 2 versus 20 s (data not shown) did show that in arctic NCMA 2099 at 2 °C NPQ relaxed somewhat slower than the temperate NCMA 1646 growing at 20 °C (ex. compare Fig. 2a with Fig. 2b), but over scales of 300 s or more, relaxation kinetics were comparable. The arctic NCMA 2099 thus retains the regulatory flexibility to induce and then relax NPQ under fluctuating light.

**Fig. 8:**
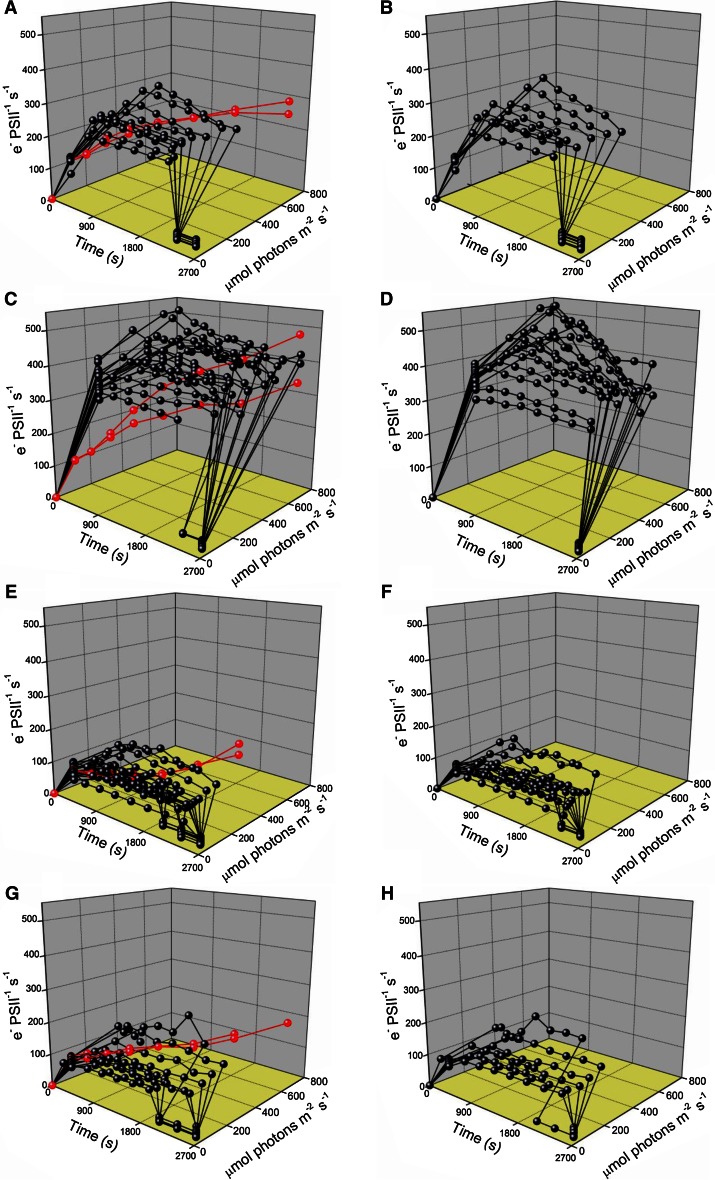
2D Plots of PSII_ETR_ without and with lincomycin. *Black symbols* represent time course data, with data from individual time courses at a particular treatment light linked by connecting lines. *Red symbols* show light response curve data, with individual light response curves again linked by connecting lines. In the time course experiments, samples were initially measured after dark incubation (*t*_0_ point). They were then exposed to the respective culture growth light (first point in time course) and then held at a treatment light for 4–7 × 300 s with an FRR measurement at the end of each 300-s interval (Fig. 2). Finally, they were shifted back to a low recovery light level of 12 μmol photons m^−2^ s^−1^ for a final 1–3 × 300 s, shown by the sharp drop in PSII_ETR_ toward the end of the time course. **a** Temperate *Micromonas* NCMA 1646 grown at 20 °C and 20–36 μmol photons m^−2^ s^−1^ without lincomycin. **b** Temperate *Micromonas* NCMA 1646 grown at 20 °C and 20–36 μmol photons m^−2^ s^−1^ with lincomycin. **c** Temperate *Micromonas* NCMA 1646 grown at 20 °C and 185 μmol photons m^−2^ s^−1^ without lincomycin. **d** Temperate *Micromonas* NCMA 1646 grown at 20 °C and 185 μmol photons m^−2^ s^−1^ with lincomycin. **e** Arctic *Micromonas* NCMA 2099 grown at 2 °C and 20–36 μmol photons m^−2^ s^−1^ without lincomycin. **f** Arctic *Micromonas* NCMA 2099 grown at 2 °C and 20–36 μmol photons m^−2^ s^−1^ with lincomycin. **g** Arctic *Micromonas* NCMA 2099 grown at 10 °C and 20–36 μmol photons m^−2^ s^−1^ without lincomycin. **h** Arctic *Micromonas* NCMA 2099 grown at 10 °C and 20–36 μmol photons m^−2^ s^−1^ with lincomycin

In Fig. 8, we summarize the function of Photosystem II in the two strains by plotting the light and time response for PSII electron transport (PSII_ETR_) (Suggett et al. 2003, 2009; Huot and Babin 2010), in the absence (Fig. 8a, c, e, g) and presence (Fig. 8b, d, f, h) of lincomycin to show the influence of PSII repair on short-term maintenance of PSII_ETR_ across the strains and growth conditions. With cumulative time and photon dose, PSII repair has a detectable influence on PSII_ETR_ in the temperate NCMA 1646, after growth at 20–36 μmol photons m^−2^ s^−1^ (compare Fig. 8a, no lincomycin, with 8B, with lincomycin), and a bigger influence after growth at 185 μmol photons m^−2^ s^−1^ (compare Fig. 8c, no lincomycin, with Fig. 8d, with lincomycin). Ongoing PSII repair was thus a significant factor to maintain PSII_ETR_ during higher illumination treatments for the temperate NCMA 1646. In the arctic NCMA 2099, PSII_ETR_ at a given light level was much lower because PSII closure (Fig. 5k, l) imposed by slow electron transport away from PSII (Table 3, *τ*_1_, *τ*_2_) and strong induction of NPQ (Fig. 5i, j) limit PSII_ETR_. In arctic NCMA 2099 after growth at 2 or 10 °C, PSII repair was a minor to negligible factor in maintaining this limited PSII_ETR_ over the course of a 2700-s high-light challenge. Instead, PSII closure and non-photochemical quenching were the dominant influences on PSII_ETR_. To be fair, for the arctic NCMA 2099 the highest light treatment levels were equivalent to levels at which PSII repair was just manifesting as significant in the temperate NCMA 1646 (Compare Fig. 8e–h with Fig. 8a–d). But at yet higher light levels, complete PSII closure and strong non-photochemical quenching meant near complete suppression of variable chlorophyll fluorescence signals in the arctic NCMA 2099, rendering any effect of instantaneous PSII repair functionally negligible for instantaneous PSII_ETR_.

## Discussion

The arctic strain under low light at 2 or 10 °C showed a classic excitation pressure acclimatory response (Huner et al. 1998) with chl b/a ratios and effective absorbance cross section for PSII photochemistry (*σ*_PSII_) both comparable to the temperate NCMA 1646 growing under much higher light (Table 1). Interestingly in the arctic NCMA 2099 at 10 **°**C, near the upper temperature limit for this psychrophile (Lovejoy et al. 2007), fitted k_recinact_ decreased, albeit with poorly constrained confidence intervals, reflecting scatter among the repeated time courses. Nevertheless, this possible decrease in functional k_recinact_ in at least some culture replicates grown at supra-optimal temperatures is consistent with the decrease in *F*_V_/*F*_M_ reflecting sustained photoinhibition (Table 2) under this growth condition. We observed similar patterns in marine diatoms (Wu et al. 2012) grown across temperature ranges, with k_rec_ peaking at the optimal growth temperature for the species, rather than showing a Q_10_-type response with increasing temperature.

Literature values for ribosomal translation rates (Guet et al. 2008) suggest that the rate constant for translation of PsbA protein is on the order of 2 × 10^−2^ s^−1^, orders of magnitude larger than the measured rate constants for PSII repair or PsbA clearance in our study, indicating that PsbA protein clearance is likely the rate limiting step upon PSII repair in these organisms. The arctic strain in particular suffered severe limitation on their clearance of PsbA protein and functional repair of PSII during light treatments (Fig. 6b; triangles). In Fig. 6c, we show that the arctic strain compensates by increasing the ratio of PsbA/[PSII]_active_, investing in reserve pools of excess PSII subunits (Behrenfeld et al. 1998) to support some PSII repair even when slow clearance of inactivated protein lags behind photoinactivation. Thus, the two strains balance their PSII repair cycles differently. The arctic strain maintains a large reserve of PSII subunits to compensate for restricted protein clearance, even under moderate growth light conditions. In parallel, the arctic strain accumulates high levels of FtsH protease subunits, possibly to partially counter kinetic limitations on protein turnover at low temperature. In studies of marine diatoms (Wu et al. 2011, 2012; Campbell et al. 2013) and currently in some marine picocyanobacteria (Cockshutt et al., unpub.), we are finding evidence for sustained presence of significant pools of [PSII]_inactive_ under various growth conditions, prior to the onset of any higher light treatment. We suggest that our modified integral equation for fitting k_recinact_ will prove generally useful for analyzing PSII repair in marine phytoplankters.

In the arctic strain *Micromonas* NCMA 2099, instantaneous repair of PSII had only a marginal influence on the maintenance of PSII_ETR_ under an upward light shift since PSII closure and strong induction of non-photochemical quenching suppressed PSII_ETR_. Under growth at 2 °C, arctic *Micromonas* NCMA 2099 maintains a constitutive induction of xanthophyll deepoxidation, shown by second-derivative whole-cell spectra, which supports a strong induction of non-photochemical quenching under low to moderate light, that does not depend upon sustained xanthophyll cycling and thus can function even if enzymatic activities are restricted at low temperature. This NPQ, however, can relax during subsequent darkness with kinetics almost comparable to the temperate *Micromonas* NCMA 1646, thereby limiting the opportunity cost of sustained down regulation of PSII function after a decrease in light (Raven 2011).

The temperate strain, in contrast, uses rapid protein clearance to maintain almost all of its PSII protein in the form of [PSII]_active_ under growth conditions, thereby using faster kinetics to achieve a better functional return on standing protein investment, with much less dependence upon induction of NPQ under physiologically relevant light intensities. It remains to be seen whether the distinct Photosystem II maintenance strategies of arctic and temperate *Micromonas* will be mirrored in other psychrophile/temperate taxon pairings (Jungblut et al. 2009; Lovejoy et al. 2011; Dolhi et al. 2013).

## Abbreviations

DTT: Dithiothreitol
